# Recurrent peripheral ischemia following endovascular repair of an infrarenal aortic aneurysm: what did we miss?

**DOI:** 10.1093/jscr/rjae823

**Published:** 2025-01-09

**Authors:** Laith A Ayasa, Anas Odeh, Saad Abuzahra, Fatima Abd Aljalil, Ahmad Qozat

**Affiliations:** Faculty of Medicine, Al Quds University, Mount of Olives Street 26, Sheikh Jarrah, PO Box 22246, Jerusalem 91513, Palestine; Department of Medicine, Faculty of Medicine and Health Sciences, An-Najah National University, PO Box 7, Nablus, Palestine; Department of Medicine, Faculty of Medicine and Health Sciences, An-Najah National University, PO Box 7, Nablus, Palestine; Cleveland Clinic Fairview Hospital, 18101 Lorain Ave, Cleveland, United States; Vascular Surgery Department, Bonifatius Hospital, Wilhelmstraße 13, 49808 Lingen, Germany

**Keywords:** abdominal aortic aneurysm, endovascular aneurysm repair, acute limb ischemia, recurrent embolization, atheromatous plaque

## Abstract

We document a case of a 75-year-old patient with a history of hypercholesterolemia and hypertension, who underwent endovascular aortic repair (EVAR) for an infrarenal abdominal aortic aneurysm (AAA) with common iliac artery ectasia. Despite an initially successful procedure, the patient experienced recurrent episodes of acute limb ischemia in his right leg. Subsequent imaging revealed thrombus formation distal to the stent graft, constituting a potential source of embolization, which warranted a reevaluation of the treatment strategy. This case highlights some of the complexities associated with managing AAA patients. In the context of EVAR, it emphasizes the significance of careful patient selection, meticulous endograft implantation, and watchful follow-up while tailoring treatment according to individual patient needs and anatomical considerations.

## Introduction

The global prevalence of abdominal aortic aneurysm (AAA) among persons aged 30–79 years is 0.92% (translating to a total of 35.12 million) [1], and with age, the risk of AAA increases significantly; it affects roughly 4–7% of males and 1–2% of women over the age of 65 [2]. Even with a rapid surgical repair, rupture is linked to high mortality. The treatment approach for AAAs has significantly evolved in the past decade. Since the initial publication by Parodi et al. in 1991 [3], endovascular repair has emerged as a viable treatment option for aortoiliac aneurysms in selected patients. This minimally invasive approach is particularly beneficial for elderly and high-risk patients who may not be suitable candidates for traditional open aneurysm repair. Several randomized controlled trials have reported lower short-term mortality rates after endovascular aortic repair (EVAR) compared to conventional open aortic repair, leading to a continuous increase in the number of EVAR procedures. Currently, EVAR is used in ~65% of all intact AAA repairs and 30% of all rupture AAA repairs [4]. While most comparative studies focus on major outcome parameters such as mortality rates, some outcomes, such as acute lower extremity ischemia (LEI), after AAA repair are less reported in the literature. LEI after AAA repair is considered a serious complication and remains one of the most challenging emergencies in vascular surgery. In this context, we present a case of a 75-year-old patient who developed recurrent episodes of acute limb ischemia following endovascular repair of an infrarenal AAA, highlighting the complexities involved in managing such cases.

## Case presentation

**Figure 1:**
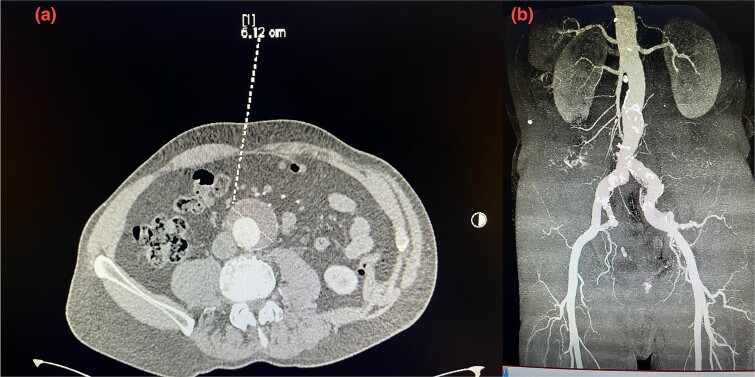
(a) Pre-operative axial CT scan of the infrarenal aorta showing an aneurysm (arrow) measuring 6.10 cm. (b) Initial CTA scan. A detailed anatomy of the bilateral iliac arteries is presented, and the axial image of the CTA demonstrated bilateral common iliac artery ectasia, measuring 2.9 cm on the right side and 2.4 cm on the left side.

A 75-year-old patient presented with recurrent episodes of acute limb ischemia described as burning pain and pallor in the right leg. The patient’s medical history included hypercholesterolemia, hypertension, a 20-pack-year smoking history**,** and no significant surgical history prior to the AAA diagnosis. He was prescribed aspirin 325 mg and atorvastatin 40 mg for hypercholesterolemia and cardiovascular disease prevention; however, the patient was non-adherent to these medications. Lipid profile at the time of presentation showed increased cholesterol levels, with a low-density lipoprotein (LDL) of 190 mg/dL, HDL of 42 mg/dL, and triglycerides of 250 mg/dL. He was transferred to our hospital with a diagnosis of an AAA measuring 6.12 cm. The aneurysm had a healthy 2 cm infrarenal neck and bilateral common iliac artery ectasia, with the right common iliac artery measuring 2.9 cm and the left measuring 2.4 cm (Fig. 1a and b). Computed tomography (CT) imaging also revealed a 5 cm atheromatous plaque at the hypogastric arteries.

**Figure 2:**
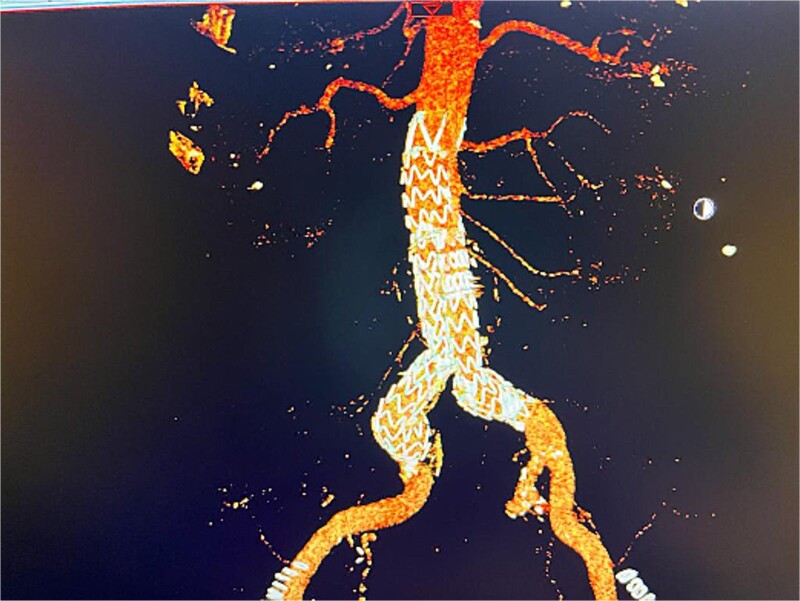
Postoperative 3D reconstruction CTA image of the EVAR without any sign of endo leak.

Given the anatomical complexity and the risk of injury to the hypogastric artery, we opted for EVAR, along with a flared limb extension to address the common iliac artery ectasia (Fig. 2). However, the patient experienced recurrent episodes of acute limb ischemia postoperatively, necessitating a thorough diagnostic and therapeutic approach.

**Figure 3:**
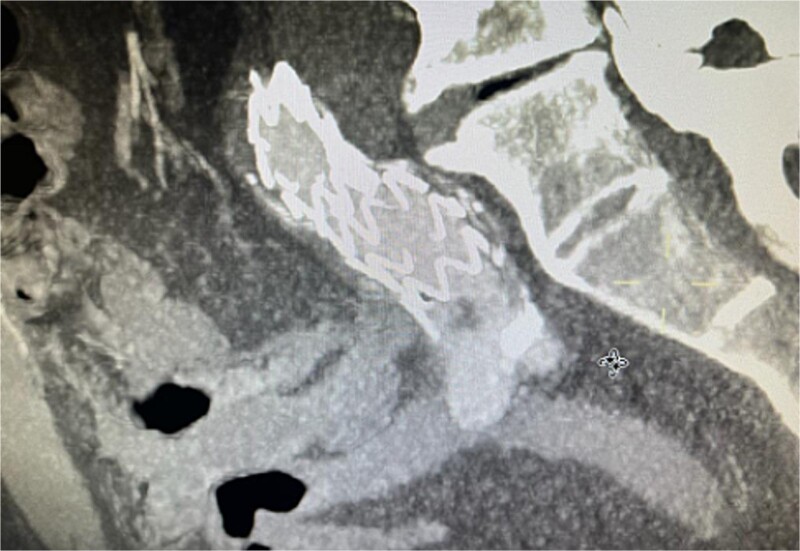
CTA scan showing the floating thrombus distal to the right iliac artery stent, following the patient presenting with acute embolic ischemia in the right leg.

The patient presented 2 months after the initial procedure to the emergency department with burning pain and pallor in his right leg. On physical examination, there was pallor below the knee in addition to loss of pulsation. Physical examination revealed loss of pulsation below the knee. CT angiography (CTA) showed total occlusion of the popliteal artery without any endoleaks, prompting an urgent transpopliteal embolectomy. Trans-esophageal Echocardiography and CTA of the aorta were performed to rule out other sources of embolization, revealing a thrombus adherent to the wall distal to the stent graft (Fig. 3). The patient was started on warfarin.

**Figure 4:**
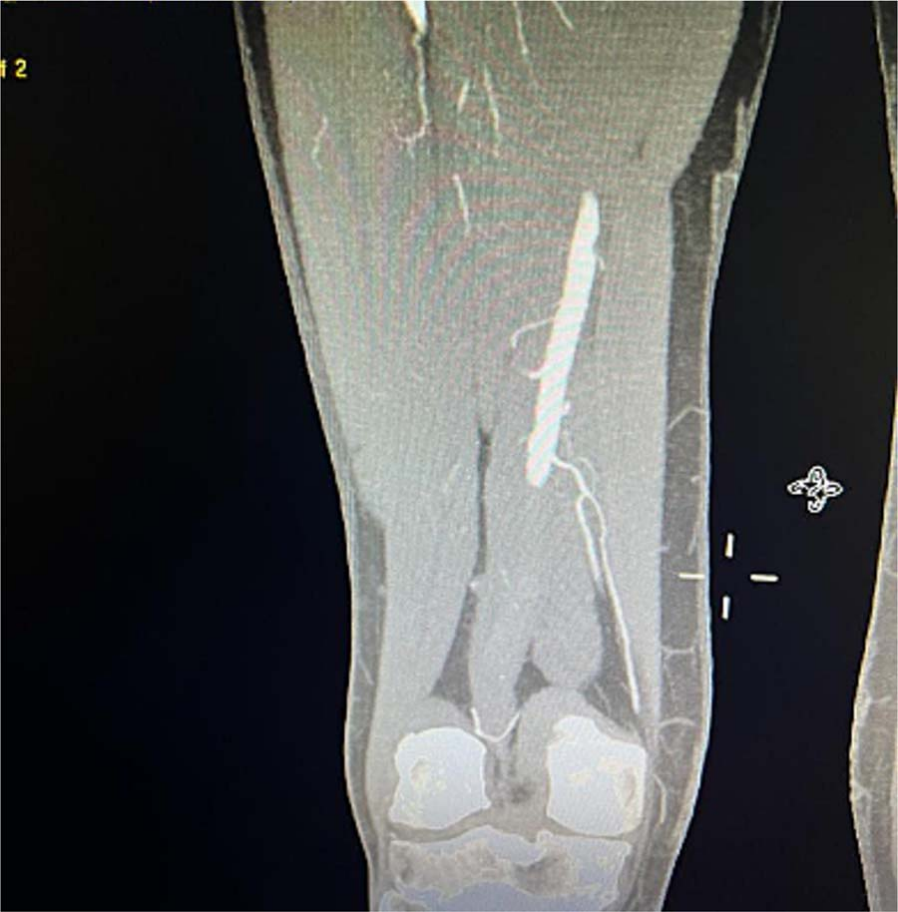
Coronal view of CTA scan showing right femoral artery embolic obstruction

**Figure 5:**
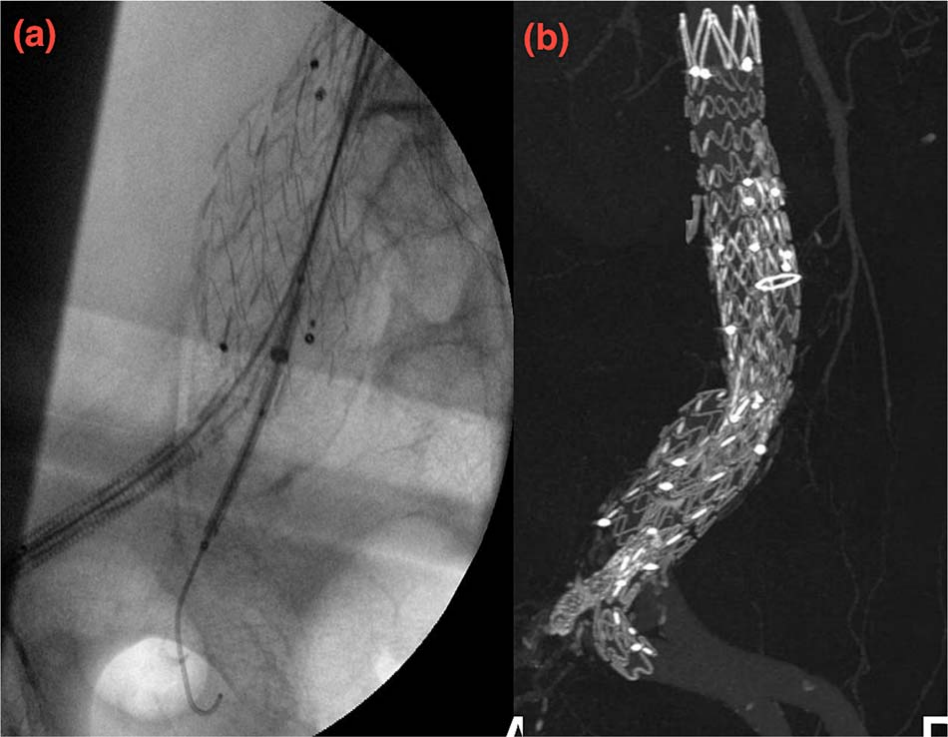
(a, b) Intraoperative images showing implantation of the iliac side branch device ISB.

Despite these interventions, the patient experienced further episodes of acute limb ischemia (Fig. 4), leading to thrombolytic therapy a month later. This recurrence prompted a reevaluation of the treatment strategy. We considered extending the graft limb, which would involve sacrificing the hypogastric artery, or using an iliac artery extension with an iliac side branch. We ultimately chose the latter and implanted an E-iliac stent graft (Jotec ISB system) (Fig. 5a and b). Preservation of the hypogastric artery was prioritized, although extending into the external iliac artery would have been a viable alternative. However, the multidisciplinary team decided the preservation of the hypogastric artery would be more appropriate in this patient. The patient was discharged without anticoagulants.

We believed the atheroma distal to the flared limb was the source of acute ischemia and subsequent embolization events. After 1 year of follow-up, the patient showed sustained improvement. Our treatment strategy focused on preserving the internal iliac artery while excluding disease in the common iliac artery using the iliac side branch system.

## Discussion

AAA is a life-threatening condition characterized by focal dilation of the abdominal aorta, affecting ~4.8% of individuals over the age of 65, with prevalence peaking in the seventh and eighth decades of life. Its pathogenesis involves permanent remodeling of the arterial wall connective tissue and is associated with risk factors such as atherosclerosis, smoking, advanced age, male gender, and white race. AAA is often asymptomatic and discovered incidentally, leading to high mortality and morbidity rates when they rupture. Therefore, ultrasound screening is recommended for male patients aged 65–75 with a history of smoking [5, 6].

Management of AAA depends on size, symptoms, and progression. Ruptured aneurysms require immediate surgical intervention, while elective surgery is considered for symptomatic aneurysms or those ≥5.5 cm. Surgical repair can be via open surgery or EVAR, with the choice depending on patient anatomy and resources. EVAR is often preferred for high-risk patients, while open surgery may offer long-term benefits for younger individuals [7, 8]. EVAR’s success relies on proper patient selection and anatomical matching of the endograft, with important criteria including aortic neck size and iliac artery diameter. Complications, such as endoleaks, stent migration, and ischemic events, occur in a subset of patients, with lower limb ischemia affecting ~9% of cases. Treatment options like the iliac branch devices (IBD) have emerged to manage complex iliac anatomy and preserve the hypogastric artery, avoiding complications like gluteal necrosis [9–11]. Treatment of aortoiliac aneurysms has evolved, with options like the bell-bottom technique and IBD being used to manage complex iliac anatomy and avoid complications such as pelvic ischemia, which can result from hypogastric artery occlusion. Sacrificing the hypogastric artery, necessary in some EVAR procedures, can lead to complications such as gluteal necrosis or buttock claudication [12, 13]. In this case, we preserved the hypogastric artery in line with Society for Vascular Surgery guidelines, which recommend maintaining at least one hypogastric artery to prevent complications like pelvic ischemia. This approach allowed us to preserve pelvic blood flow and reduce the risk of recurrent limb ischemia, emphasizing the importance of personalized treatment strategies [14].

## Conclusion

Our case highlights the importance of an adaptable strategy when faced with complex aortoiliac anatomies in EVAR patients, demonstrating the need for careful evaluation of all possible treatment options. By opting for the iliac side branch system, we were able to preserve critical blood flow to the pelvis, avoid unnecessary sacrifice of the hypogastric artery, and successfully mitigate the risk of recurrent limb ischemia. This case emphasizes the necessity of personalized intervention strategies to ensure optimal outcomes in such high-risk patients.
